# A big data-based prediction model for prostate cancer incidence in Japanese men

**DOI:** 10.1038/s41598-023-33725-8

**Published:** 2023-04-21

**Authors:** Mineyuki Kato, Go Horiguchi, Takashi Ueda, Atsuko Fujihara, Fumiya Hongo, Koji Okihara, Yoshinori Marunaka, Satoshi Teramukai, Osamu Ukimura

**Affiliations:** 1 Department of Urology, Graduate School of Medical Science, Kyoto Prefectural University of Medicine, Kyoto-City, Kyoto 602-8566 Japan; 2 Department of Biostatistics, Kyoto Prefectural University of Medicine, Kyoto-City, Kyoto Japan; 3 Department of Urology, North Medical Center Kyoto Prefectural University of Medicine, Yosano-gun, Kyoto, Japan; 4 Medical Research Institute, Kyoto Industrial Health Association, Kyoto, Japan

**Keywords:** Cancer, Cancer epidemiology, Diagnostic markers

## Abstract

To define a normal range for PSA values (ng/mL) by age and create a prediction model for prostate cancer incidence. We conducted a retrospective analysis using 263,073 observations of PSA values in Japanese men aged 18–98 years (2007–2017), including healthy men and those diagnosed with prostate cancer. Percentiles for 262,639 PSA observations in healthy men aged 18–70 years were calculated and plotted to elucidate the normal fluctuation range for PSA values by age. Univariable and multivariable logistic regression analyses were performed to develop a predictive model for prostate cancer incidence. PSA levels and PSA velocity increased with age in healthy men. However, there was no difference in PSA velocity with age in men diagnosed with prostate cancer. Logistic regression analysis showed an increased risk of prostate cancer for PSA slopes ranging from 0.5 to 3.5 ng/mL/year. This study provides age-specific normal fluctuation ranges for PSA levels in men aged 18–75 years and presents a novel and personalized prediction model for prostate cancer incidence. We found that PSA slope values of > 3.5 ng/mL/year may indicate a rapid increase in PSA levels caused by pathological condition such as inflammation but are unlikely to indicate cancer risk.

## Introduction

The prostate is a gland in the male reproductive system, and prostate-specific antigen (PSA) is a serine protease synthesized in the prostate epithelium and mainly secreted into seminal fluid^1^. The expression of the PSA gene (i.e., *KLK3*) is regulated by androgen levels through androgen receptor (AR) signal activation^1^. In healthy men with normal prostate, only a portion of PSA is secreted into the blood, and blood PSA levels (in ng/mL) can fluctuate according to age; this partly reflects differences in prostate volume. Meanwhile, in men with prostate cancer (i.e., where AR signaling is highly activated), PSA is expressed at higher than expected levels in reference to prostate volume, resulting in increased blood PSA levels.

Natural PSA fluctuation in healthy men according to age remains unclear. Previously, several studies have reported a natural progression of increased PSA levels with age in healthy men; however, these studies have enrolled modest sample sizes^2–4^. In addition, a study enrolling 90,000 Brazilian men under 40 years of age reported slow increases in PSA levels in this age group^5^. Large-scale investigations evaluating PSA measurements in men over the age of 40 years are needed to understand natural PSA kinetics. These results have the potential to provide useful information in the clinical application of PSA results by defining normal fluctuation ranges for PSA levels by age.

Studies report that PSA-based screening for prostate cancer reduces the risk of death due to prostate cancer^6,7^. Since normal age-related PSA fluctuation is caused by normal changes in prostate volumes / the normal aging process and by inflammation and benign prostate disease (such as benign prostate hyperplasia; BPH)^8^, a single measurement of blood PSA levels may result in false-positive findings^9^. Calculating the PSA change rate based on PSA data taken at two or more time points may be one potential solution for overcoming this issue.

Currently, there are two major suggested methods for calculating changes in PSA levels for the purpose of predicting prostate cancer incidence^10^. One method is to calculate PSA velocity (in ng/mL/year), representing the average rate of change in PSA values over time. The other method is to calculate the PSA slope (ng/mL/year) using linear regression modeling. PSA slope has shown better sensitivity and specificity than PSA velocity in the prediction of prostate cancer incidence within prior research^11^.

Although several studies regarding the clinical use of PSA velocity and PSA slope in predicting prostate cancer incidence have been reported (since initial findings reported by Carter et al.^13^), several issues have not yet been addressed. First, to our knowledge, fluctuation ranges that account for inflammation has not been calculated. More specifically, although a rapid increase in blood PSA levels is known to be attributable to inflammation^8,14^, the fluctuation range of PSA blood levels according to inflammation has not been investigated in previous studies. Second, the effects of age on the results of PSA velocity findings are presently unclear. While Loeb et al. reported that PSA velocity in both healthy men and patients with prostate cancer aged ≥ 50 years was not affected by age, Moul et al. suggested that PSA velocity in both healthy men and patients with prostate cancer aged 50–59 years was lower than that in patients aged 70 years or older^15,16^. Investigation regarding the two abovementioned issues may lead to a more precise prediction of prostate cancer incidence according to changes in PSA values.

The aims of the present study were as follows. First, we aimed to obtain a natural history of PSA and PSA velocity in healthy men (i.e., men without prostate cancer) using big data on PSA measurements (i.e., over 260,000 occurrences) in Japanese men within a wide, representative age range. We also aimed to identify a prediction model for prostate cancer incidence according to PSA slope and age, excluding PSA fluctuation occurring due to pathological condition such as inflammation.

## Methods

A total of 263,073 observations of PSA values in men aged 18–98 years (including men with prostate cancer) taken between 2007 and 2017 were obtained from the Kyoto Industrial Health Association, an organization that performs medical health examinations (including PSA screening tests) in Kyoto, Japan. Prostate cancer was diagnosed and treated at other hospitals. In addition, blood PSA levels were measured using a chemiluminescent immunoassay system (Advia Centaur, Siemens Healthcare Diagnostics, USA).

This study was approved by the Institutional Review Board affiliated with the Kyoto Industrial Health Association and the Kyoto Prefectural University of Medicine (Approval No. ERB-C-1071-1). The institutional review board waived informed consent due to the retrospective nature of this investigation. Opt-out information was provided to participants on the Kyoto Prefectural University of Medicine website. This work was conducted following the principles of the Declaration of Helsinki.

PSA plots showing 25th, 50th, 75th, 90th, and 95th percentile PSA values (n = 262,639) for healthy men were calculated by age in order to investigate natural PSA fluctuation trajectories. Fluctuation in PSA velocity (i.e., the average rate of change in PSA over time) was calculated using PSA values for 35,140 healthy men and 125 men diagnosed with prostate cancer who underwent multiple tests for more than two years in a row.

### Study population

We chose to implement the PSA slope (ng/mL/year) calculated using linear regression in creating a prediction model for prostate cancer incidence, as PSA slope had shown better sensitivity and specificity than PSA velocity in previous studies^11^. Since calculating the PSA slope requires data on PSA values taken at multiple time points; the population used to develop the prediction model was extracted as follows. First, a reference time point was established for each subject for PSA data measured at one-year intervals. The reference time point was defined as either the time when the subject was determined to have cancer or the time when PSA was last measured (Fig. S1). Next, for each subject, we checked PSA measurement data up to three years back from the reference time point and selected subjects for whom PSA values were measured at two or more of the three available time points.

### Statistical analysis

In regard to the subjects' demographic characteristics, we calculated means and standard deviations (SD) for continuous variables and calculated numbers and percentages for categorical variables. To assess the degree of overfitting of the predictive model, the dataset was stratified by the presence or absence of prostate cancer and randomly split into two (training set: validation set = 7:3) and the following analyses were performed. We performed univariable and multivariable logistic regression analyses using the training set to develop a model predicting the incidence of prostate cancer one year after the measurement time point. Predictors included age, PSA slope, and the avarage PSA value (i.e., the average PSA measurement taken at two or three-time points). Age was defined as the age at the closest measurement time point with the available data relative to the reference time point.

To confirm the linearity of the association between PSA slope and prostate cancer risk, we performed a logistic regression analysis using the training set with PSA slope (defined according to 0.5 increments) as the independent variable and age and the mean PSA value as covariates. Then, we plotted the regression coefficient for the PSA slope.

We evaluated the prediction model's performance in terms of discrimination and calibration; we calculated the area under the receiver operating characteristic curve (AUROC) using the training and validation sets to evaluate discrimination performance. We also evaluated calibration using a calibration plot and the Hosmer–Lemeshow test. Decision curve analysis^12^ was also performed to evaluate the clinical utility of the developed prediction model.

All reported P-values were two-sided, with P-values of < 0.05 indicating statistical significance. Statistical analyses were performed using SAS statistical software (v.9.4, SAS Institute Inc., Cary, NC, USA.).

## Results

An age hierarchy histogram for a cumulative total of 263,073 PSA value observations is shown in Fig. S2. This study enrolled 78,407 men; 22,419 men underwent testing only once, whereas others underwent testing multiple times. A total of 434 men (0.55%) who were clinically diagnosed with prostate cancer were included.

**Figure 1:**
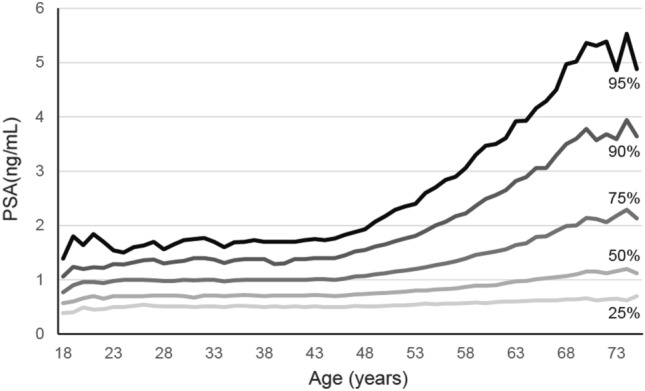
Natural PSA fluctuation by age. PSA plot showing 25th, 50th, 75th, 90th, and 95th percentiles for PSA values in healthy men by age. *PSA* prostate-specific antigen.

To observe natural PSA fluctuation by age, we constructed PSA plots showing 25th, 50th, 75th, 90th, and 95th percentile PSA values by age using 262,639 PSA measurements taken in healthy men (i.e., excluding 434 men who were diagnosed with prostate cancer) (Fig. 1). We found that PSA values were normally distributed for each age group. For example, the median PSA levels for the 40–49 year, 50–59 year, and 60–69 year age groups were 0.72, 0.80, and 0.97 ng/mL, respectively.

As shown in Fig. 1, PSA in those younger than 40 years of age showed a very slow increase, consistent with past reports^5^. In contrast, after the age of 40 years, the 50th, 75th, 90th, and 95th percentiles were found to increase statistically significantly.

In addition, we calculated PSA velocity in 35,140 healthy men and 125 men diagnosed with prostate cancer who underwent multiple tests for more than two years in a row in order to observe PSA velocity fluctuation in both healthy men and men diagnosed with prostate cancer according to age (Figs. S3 and S4). While the PSA velocity in healthy men increased after the age of 40 years, we found no statistically significant difference in PSA velocity in men diagnosed with prostate cancer in comparative evaluations between age groups.

Next, we tried to develop a new calculation method for predicting prostate cancer incidence according to PSA slope, as this parameter has been reported to have better sensitivity and specificity than PSA velocity for predicting prostate cancer incidence^11^. Of the 78,407 subjects enrolled in this study, 23,345 subjects who were evaluable according to study criteria were included in the analytic set for the development of the predictive model; we excluded 55,062 subjects with fewer than two measurement time points for PSA data relative to the reference point (i.e., three years prior; Fig. S5). In addition, the 23,345 subjects were split into a training set of 16,340 and a validation set of 7,005.

The demographic characteristics of the included subjects are shown in Table S1. The mean values (SD) for age and PSA slope for the training set were 55.2 (10.2) years and 0.03 (0.67) ng/mL/year, respectively. For the training set, an average PSA of < 1.5 ng/mL was the most common finding, with 13,148 detected findings in this range (80.5%); the number of subjects decreased with each successive PSA category. There were no large differences in the distribution of variables between the training and validation sets.

**Figure 2:**
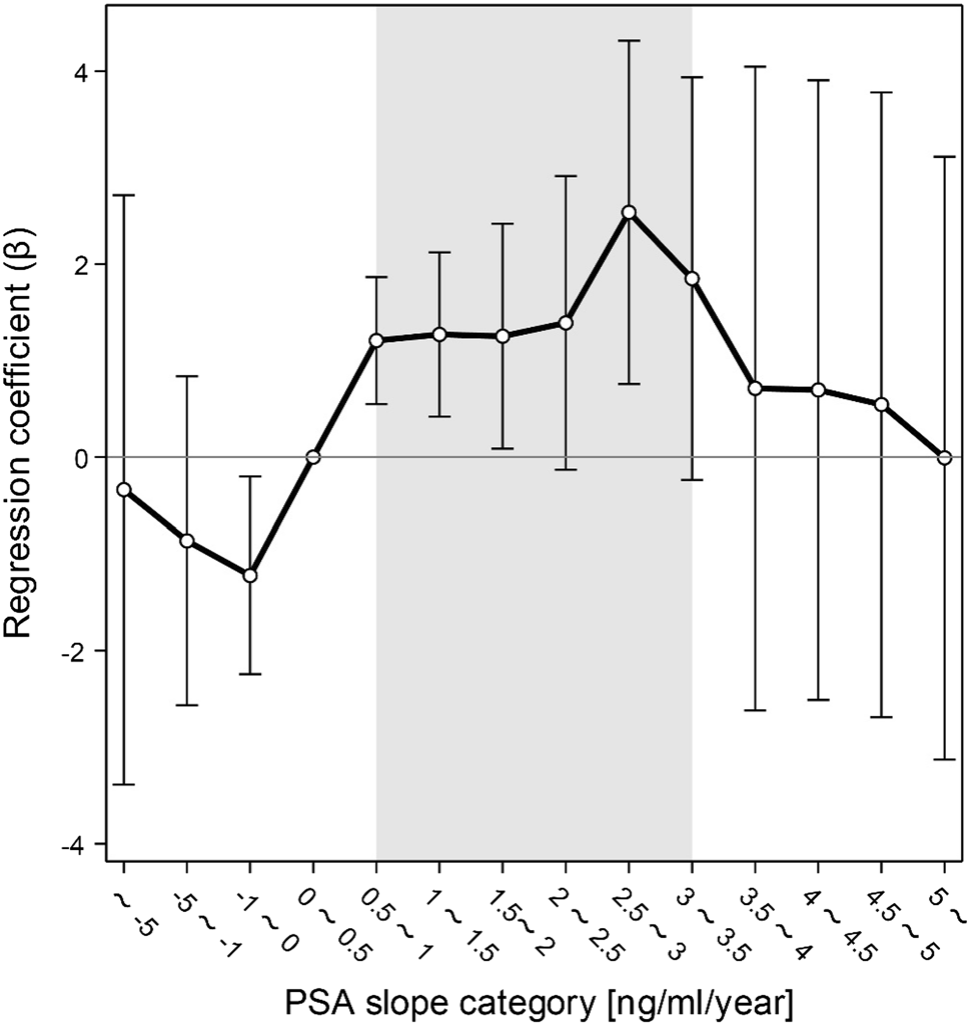
Linearity of the effect of PSA slope on the risk of prostate cancer (traning set). Logistic regression analysis was performed with PSA slope (in 0.5 increments) as the independent variable, and age and mean PSA value as covariates. We plotted the regression coefficient according to PSA slope. Our results showed an increased risk of prostate cancer for PSA slopes ranging between 0.5 and 3.5 ng/mL/year. *PSA* prostate-specific antigen.

The linearity of the effect of PSA slope on the risk of developing prostate cancer was likewise confirmed in the present study, demonstrating an increased prostate cancer risk for PSA slopes ranging between 0.5 and 3.5 ng/mL/year (Fig. 2). Therefore, the PSA slope was included in the predictive model as a binary variable (i.e., "0.5–3.5" and "other").

**Figure 3:**
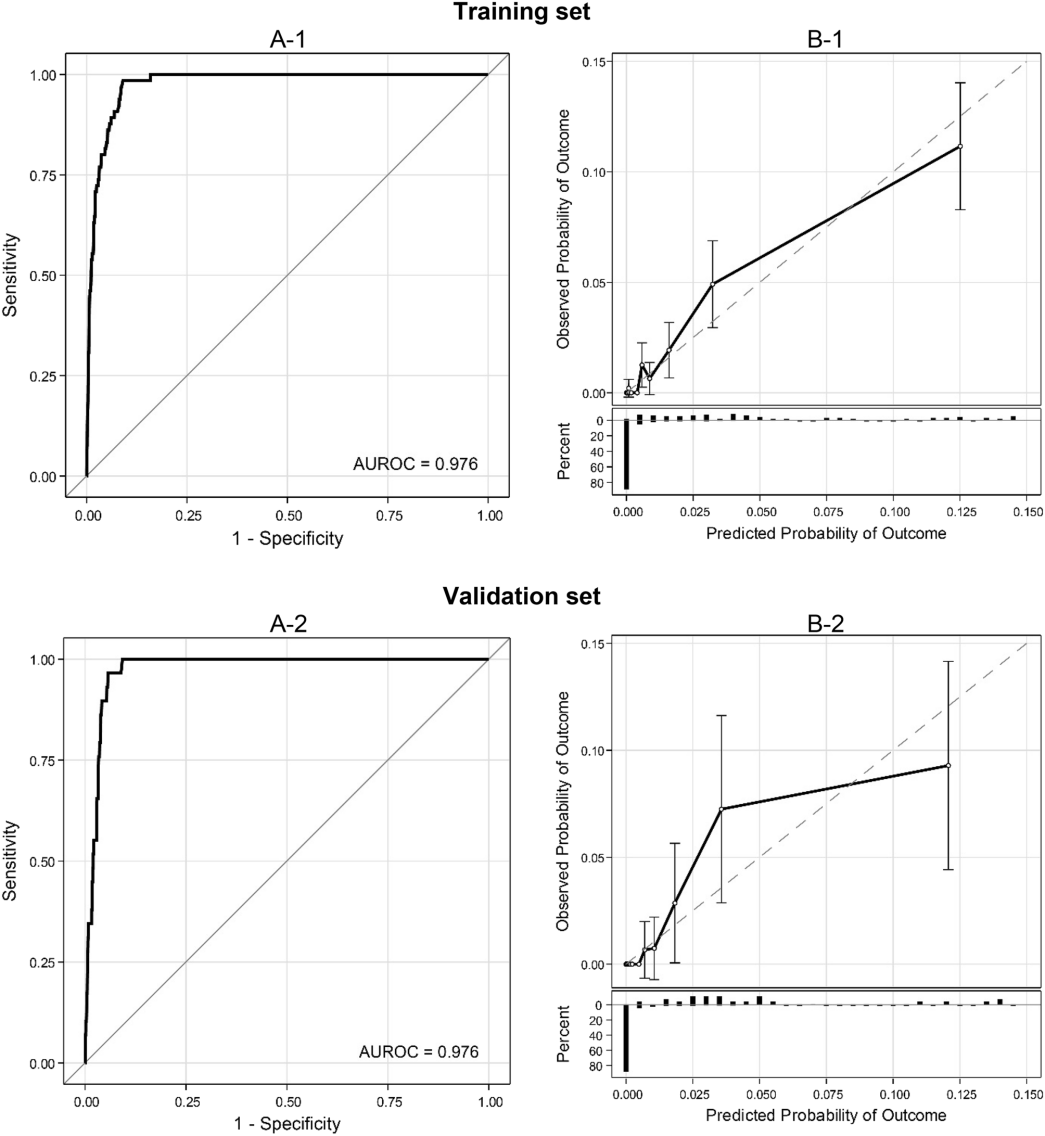
ROC curves (**A**) and calibration plots (**B**). The AUROC [95% CI] for the training and validation sets were 0.976 [0.969, 0.984] and 0.976 [0.968, 0.984], respectively. *AUROC* area under the ROC, *ROC* receiver operating characeristic.

The results of the logistic regression analysis using the training set are shown in Table 1. Multivariable analysis demonstrated that the PSA slope was statistically significantly associated with the incidence of prostate cancer (odds ratio [95% confidence interval (CI)] = 5.94 [3.41, 10.3], P < 0.001). In addition, age and average PSA value were also found to be statistically significant predictors of prostate cancer incidence. From the $\beta$-coefficients in Table 1, predicted probability of developing prostate cancer in one year is calculated as 1/(1 + exp(− X)), where X = − 6.856 + 0.055*Age + 1.782 (if 0.5 < PSA slope < 3.5) − 5.787 (if average PSA < 1.5) − 3.273 (if 1.5 ≤ average PSA < 2) − 1.601 (if 2 ≤ average PSA < 3) − 0.709 (if 3 ≤ average PSA < 4). ROC curves and calibration plots are shown in Fig. 3. The AUROC [95% CI] for the training and validation sets were 0.976 [0.969, 0.984] and 0.976 [0.968, 0.984], respectively, with little difference. For the calibration plots, there was a trend toward underestimation near 3% and overestimation near 12% of the predicted probability for the validation dataset, but the Hosmer–Lemeshow test showed that the model was well calibrated for both datasets (training set: P = 0.994, validation set: P = 0.994). We also examined models that included the PSA slope or average PSA value as a continuous variable, but both discrimination and calibration performance were inferior to the final model (Table S2). Decision curve analysis showed that the predictive model was more useful than the treat-all and treat-no-one strategies (Fig. S6).

**Table 1:** Univariable and multivariable logistic regression analysis including age, PSA slope, and the average psa value as predictors (traning set).

Variables		Cancer, n = 65	Non-cancer, n = 16,275	Univariable			Multivariable			
				Odds ratio	[95% CI]	P-value	β	Odds ratio	[95% CI]	P-value
Age	1 year	65.9 (6.5)	55.2 (10.2)	1.12	[1.09, 1.15]	< 0.001	0.055	1.06	[1.02, 1.09]	0.001
PSA slope	0.5–3.5	43 (66.1)	613 (3.8)	49.9	[29.7, 84.0]	< 0.001	1.782	5.94	[3.41, 10.3]	< 0.001
	Other	22 (33.9)	15,662 (96.2)	Ref	–	–	–	Ref	–	–
Average PSA value^a^	< 1.5	1 (1.5)	13,147 (80.8)	< 0.01	[< 0.01, < 0.01]	< 0.001	− 5.787	0.003	[< 0.01, 0.02]	< 0.001
	1.5–2	2 (3.1)	1,302 (8.0)	0.02	[0.004, 0.06]	< 0.001	− 3.273	0.04	[0.01, 0.16]	< 0.001
	2–3	12 (18.5)	1,070 (6.6)	0.11	[0.06, 0.21]	< 0.001	− 1.601	0.20	[0.10, 0.41]	< 0.001
	3–4	16 (24.6)	427 (2.6)	0.36	[0.20, 0.67]	0.001	− 0.709	0.49	[0.26, 0.93]	0.028
	≥ 4	34 (52.3)	329 (2.0)	Ref	–	–	–	Ref	–	–

Continuous variable are preented as means (standard deviations). Categorical variables are presented as no. (%).

*CI* confidence interval, *PSA* prostate-specific antigen.

^a^Average of PSA measures taken at two or three time points.

**Figure 4:**
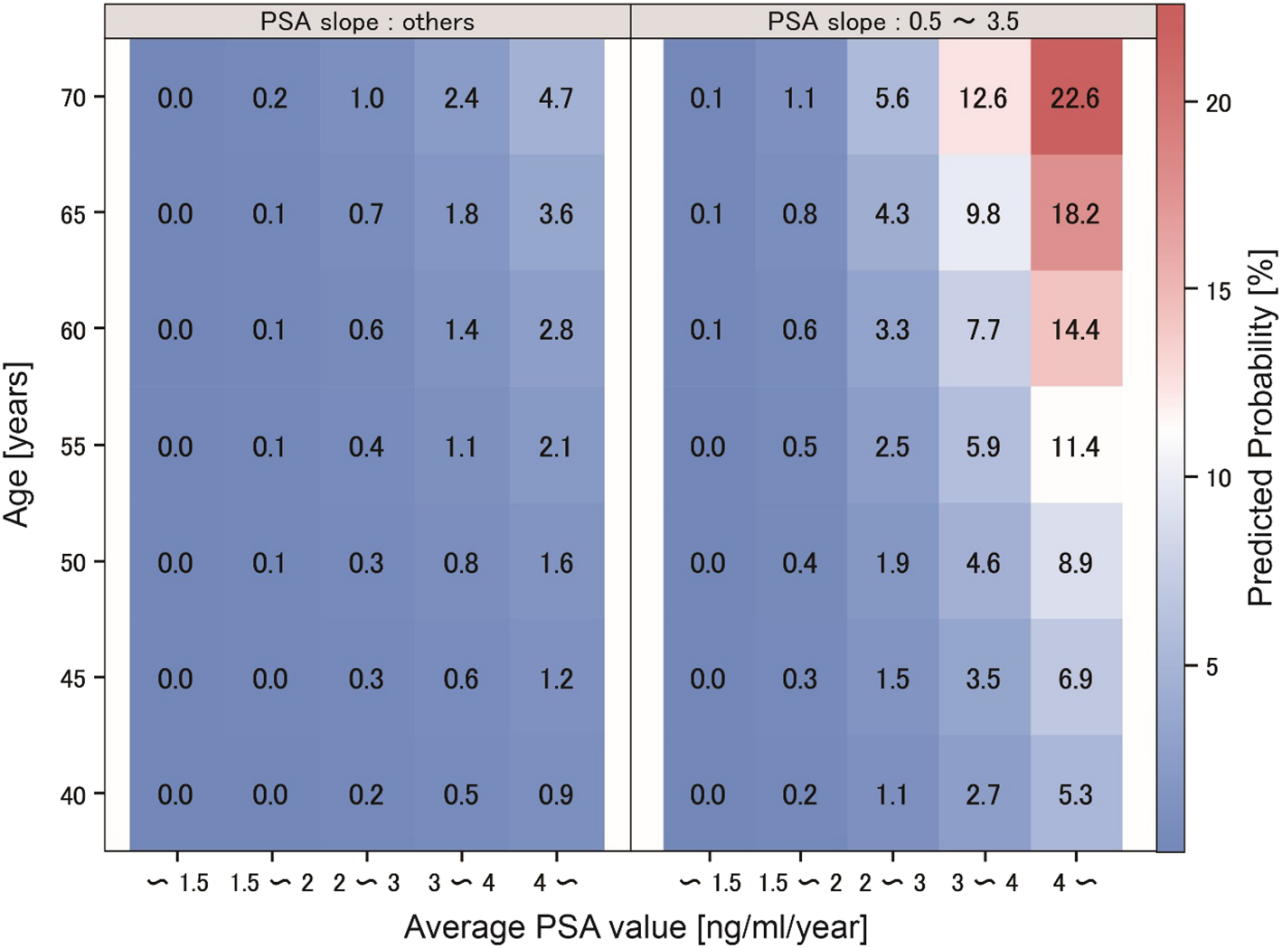
Heat map based on the developed prediction model. The heat map shows the predicted probability of developing prostate cancer in a given subject according to age, PSA slope, and average PSA value. *PSA* prostate-specific antigen.

Finally, to visualize the distribution of the predicted probability of developing prostate cancer, we created a heat map based on the constructed prediction model, as shown in Fig. 4. This figure can be used to obtain the approximate predicted probability of prostate cancer incidence for a subject according to age, PSA slope, and mean PSA value.

## Discussion

In this report, we evaluated natural PSA fluctuation by age using big data for PSA values (for a total of 263,073 observations) in a study conducted in Japan. To the best of our knowledge, our study provides the largest dataset reporting PSA values in men of all ages (focusing on those between 18 and 75 years of age). PSA data for 90,000 Brazilian men younger than 40 years of age had previously been reported in a 2018 study^5^. The normal range of PSA values by age (in all adults) provided by this study, if confirmed, would be highly useful information in clinical practice. Importantly, we developed a new prediction model including age as a continuous variable and PSA slope and average PSA as binary variables to predict prostate cancer incidence. To the best of our knowledge, this is the first report to provide a calculation tool using both PSA slope and age to predict prostate cancer incidence. The linearity of the effect of the PSA slope on prostate cancer risk was confirmed in the present study, showing an increased risk of prostate cancer for PSA slopes ranging between 0.5 and 3.5 ng/mL/year. Conversely, the probability of prostate cancer risk decreased for PSA slopes of > 3.5 ng/mL/year. Several reports showed that some patients with significantly elevated PSA level suggested prostatic inflammation by histological analysis and others with significantly elevated PSA level did not need histological analysis because of rapid decrease in PSA level by antibiotics treatment^8^. Based on the above fact, we suggest that a PSA slope value of > 3.5 ng/mL/year may indicate PSA fluctuation due to pathological condition such as inflammation. To create more sophisticated model to predict prostate cancer incidence by incorporating other covariates such as C-reactive protein (marker for inflamation) and other potential prostate cancer marker^17^ is our future work.

A uniform 4.0 ng/mL PSA threshold has long been used to detect prostate cancer (regardless of age). Since PSA increases with age, as demonstrated in this study, we conclude that the PSA threshold should instead be set separately by age group. Although the effectiveness of an age-adjusted PSA threshold has been suggested in several reports^18,19^, optimal PSA thresholds according to age have not yet been defined. Our study may be helpful in setting age-specific PSA thresholds in the prediction of prostate cancer incidence.

Several studies have shown that prostate size increases with age (i.e., starting from 40 years of age), according to imaging findings (e.g., transrectal ultrasound [TRUS] and magnetic resonance imaging [MRI] findings)^20,21^. However, changes in prostate size before 40 years of age are currently unclear, and investigating this question using the above imaging modalities is unrealistic.

PSA is expressed in an organ-specific manner, and age-related PSA changes in healthy men may be associated with normal increases in prostate size. Our research suggests that prostate size does not change until 40 years of age and then starts to increase from 40 years onwards during normal aging. Other potential explanations for changes in PSA values include the existence of BPH, clinically insignificant (i.e., latent) cancer, or undetected clinically significant cancer.

Previously, Loeb et al. reported a PSA velocity of < 0.75 ng/mL/year in healthy men and likewise reported that PSA velocity was not affected by age^16^. In contrast, our results suggest that PSA velocity increases with age (again, starting from 40 years of age). In addition, based on a previous report showing that PSA velocity differs between races^22^, we note that differences in PSA velocity between reports may be attributable to differences in race/ethnicity between study populations. On the other hand, our data suggest that PSA velocity in patients with prostate cancer range between 1.0 and 2.0 ng/mL/year regardless of age.

We note that D'Amico et al. previously reported that a PSA velocity of > 2.0 ng/mL/year before radical prostatectomy was associated with a high risk of death from prostate cancer. Nelson et al. suggested that PSA velocity was associated with clinical progression in patients with low-risk prostate cancer. We presume that the patients with prostate cancer enrolled in our study may predominantly have had relatively low-risk prostate cancer, according to our findings in regard to PSA velocity. An analysis of the association between PSA velocity and the clinical stage of enrolled prostate cancer patients is awaited in future work.

This study also suggested that baseline PSA values and PSA slope were each affected by age. Although several methods for calculating PSA change values (in order to predict prostate cancer incidence) have been suggested in previous reports^10^, to our knowledge, the influence of age has not yet been considered. Herein, we provide a novel calculation method for predicting prostate cancer incidence according to PSA slope and in consideration of age.

The Rotterdam Prostate Cancer Calculator was previously developed to predict prostate cancer incidence considering age, a single PSA value, and other radiographical findings^23^. This tool was also applied in calculating prostate cancer probability in a primary negative biopsy setting^24^. However, judging prostate cancer risk from a single PSA value could lead to both false-positive and false-negative findings, and PSA slope may be a more useful indicator in the primary negative biopsy setting. In future work, we aim to validate our model in a primary negative biopsy setting.

Although a robust increase in blood PSA values is thought to be attributed to inflammation^8,14^,, to our knowledge, there have been no reports defining the range of PSA fluctuation according to inflammation. Our study shows that a PSA slope value of > 3.5 ng/mL/year may indicate a rapid increase in PSA caused by pathological condition including inflammation but is unlikely to be due to cancer.

We acknowledge some limitations of this work. First, PSA data within a Japanese study population could reflect race-specific findings. However, Ito et al. reported no statistically significant difference in prostate cancer incidence between Dutch and Japanese men with the same baseline PSA levels^25^, and McGreevy et al. found that race did not affect changes in PSA values in an investigation evaluating comparative changes in PSA values between black and white men in the US^26^. Second, the accuracy of secondary medical examinations in diagnosing prostate cancer may differ according to the hospital; this is notable, as our patients were enrolled from multiple hospitals. In addition, we conducted a retrospective study; a prospective study, including validation of the suggested calculation method in predicting prostate cancer incidence at a single facility, is now underway.

In conclusion, this study provides age-specific normal fluctuation ranges for PSA levels in men between 18 and 75 years of age, and provides a novel and personalized prediction model for prostate cancer incidence. To the best of our knowledge, our study provides the largest dataset reporting PSA values in men of all ages (with a focus on those between 18 and 70 years of age). In addition, it is the first report to provide a calculation tool using both PSA slope and age to predict prostate cancer incidence. Our findings, if confirmed, will be useful in informing more personalized and precise screening and diagnostic protocols.

## Supplementary Information

Supplementary Information.

## Data Availability

The datasets used and analysed during the current study available from the corresponding author on reasonable request.
