# Supplementary material for: A big data-based prediction model for prostate cancer incidence in Japanese men
Source: Sci Rep. 2023 Apr 21;13:6579. doi: 10.1038/s41598-023-33725-8 (PMC10121595; doi:10.1038/s41598-023-33725-8)
Supplement: Supplementary file 1 — Supplementary Information. [file 41598_2023_33725_MOESM1_ESM.docx]

**A Big Data-based Prediction Model for Prostate Cancer Incidence in Japanese Men**

Mineyuki Kato ^1†^,MD, Go Horiguchi^2†^, MS, Takashi Ueda^1^**^*^**,MD,PhD,

Atsuko Fujihara^1^,MD,PhD, Fumiya Hongo^1^,MD,PhD, Koji Okihara^3^,MD,PhD, Yoshinori Marunaka^4^,MD,PhD, Satoshi Teramukai^2^,PhD, Osamu Ukimura^1^,MD,PhD.

^1^ Department of Urology, Kyoto Prefectural University of Medicine, Kyoto-City, Kyoto, Japan

^2^ Department of Biostatistics, Kyoto Prefectural University of Medicine, Kyoto-City, Kyoto, Japan

^3^ Department of Urology, North Medical Center Kyoto Prefectural University of Medicine, Yosano-Gun, Kyoto, Japan

^4^ Medical Research Institute, Kyoto Industrial Health Association, Kyoto, Japan

^†^These authors equally contributed to this work.

^*^ Corresponding author. Takashi Ueda,MD PhD

Address: Department of Urology, Graduate School of Medical Science, Kyoto Prefectural University of Medicine, Kyoto-City, Kyoto 602-8566, Japan

FAX: +81 75 251 5598

TEL: +81 75 251 5595

E-mail: t-ueda@koto.kpu-m.ac.jp

Key words: prostate-specific antigen, PSA velocity, PSA slope,prostate cancer, prediction model

**Supplementary Information**

**Figure S1. Analytic design of prediction model.**

The reference time point was defined as either the time when the subject was determined to have cancer or the time when PSA was last measured.

**Figure S2. Age hierarchy histogram for the cumulative total of 263,073 observations.**

The counts for PSA values in each age range are shown. PSA, prostate-specific antigen

**Figure S3. Natural fluctuation in PSA velocity by age.**

PSA velocity by age was calculated using PSA values measured in 35,140 healthy men who underwent tests for more than two years in a row. We generated PSA velocity plots showing the 25th, 50th, 75th, and 90th percentiles for this parameter by age.

**Figure S4.** **PSA velocity for men diagnosed with prostate cancer.**

PSA velocity was calculated using PSA values for 125 men diagnosed with prostate cancer who underwent tests for more than two years in a row. PSA, prostate-specific antigen

**Figure S5. Study population flowchart.**

A analytic subset of 23,345 subjects were extracted from a total of 78,407 enrolled subjects to develop the prediction model, excluding 55,062 subjects with fewer than two measurement PSA measurement time points available from the reference point to three years prior.

**Figure S6. Decision curve analysis.**

The developed prediction model was applied to the training and validation datasets and the relationship between threshold probability and Net benefit was plotted.

**Table S1.** **Demographic characteristics of patients extracted from the total sample to develop the predictive model.**

**Table S2. Results of Models including PSA slope or the Average PSA Value as Predictors of Continuous Variables (Traning set).**


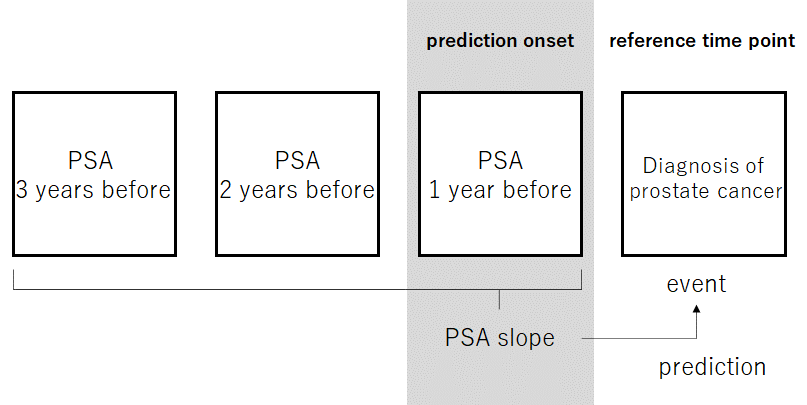
**Figure S1.**

**Figure S2. Age hierarchy histogram for the cumulative total of 263,073 observations.**


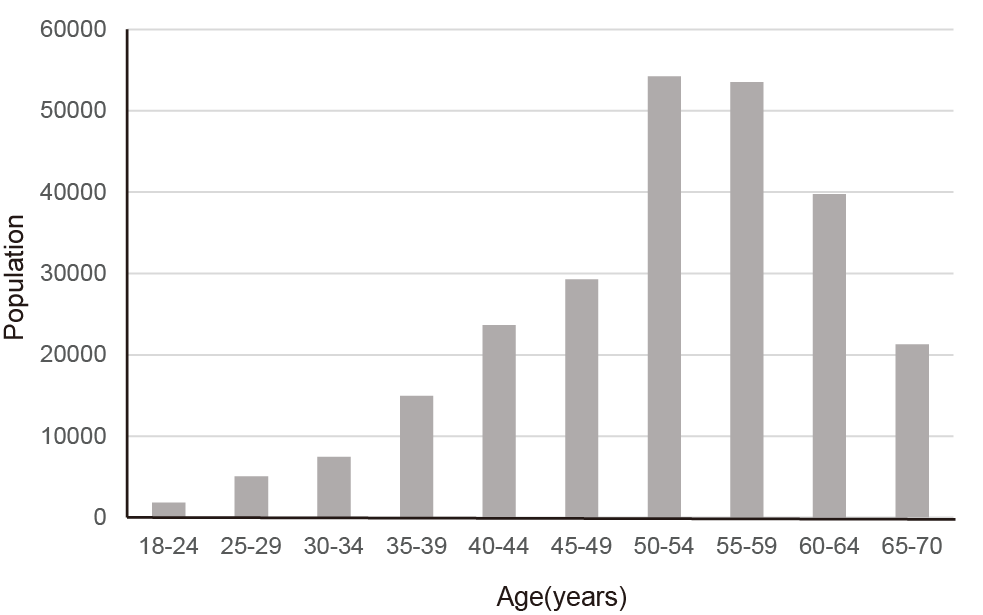
**Figure S3. Natural fluctuation in PSA velocity by age.**


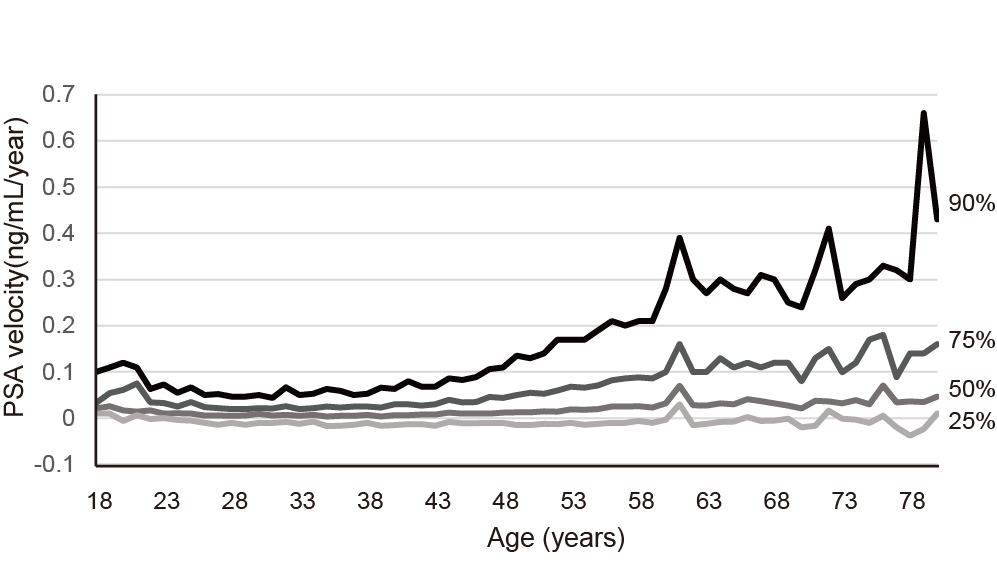


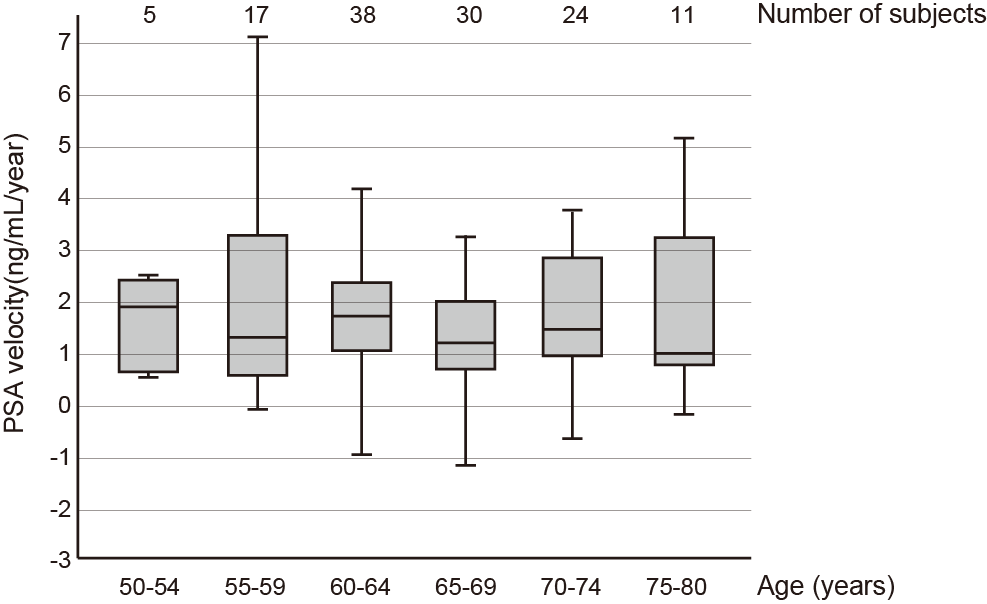
**Figure S4. PSA velocity for men diagnosed with prostate cancer.**

**Figure S5. Study population flowchart.**

**Figure S6. Decision curve analysis.**

**Traning set**

**
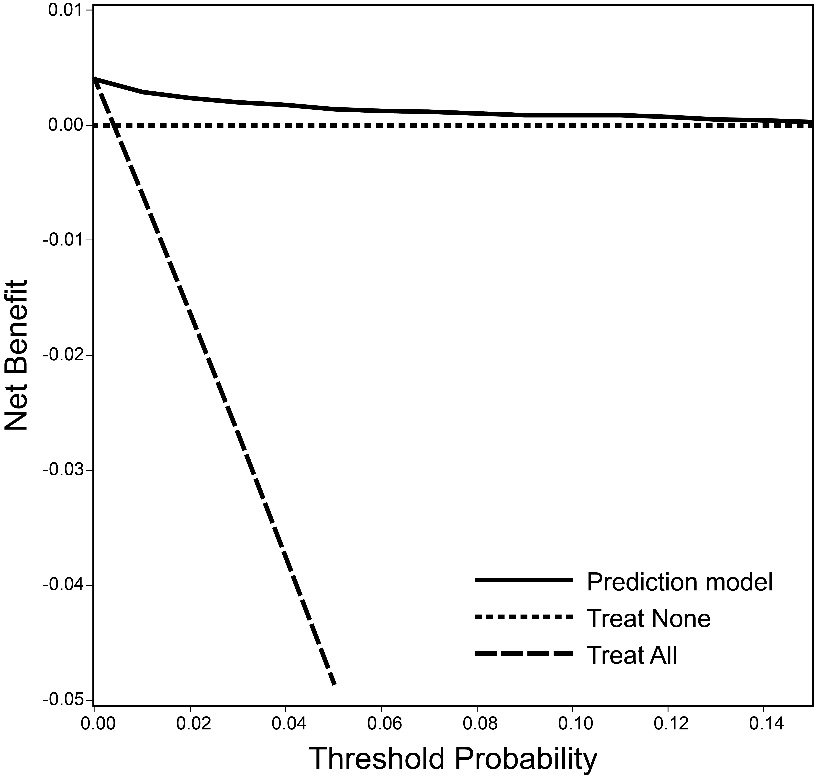
**

**Validation set**

**
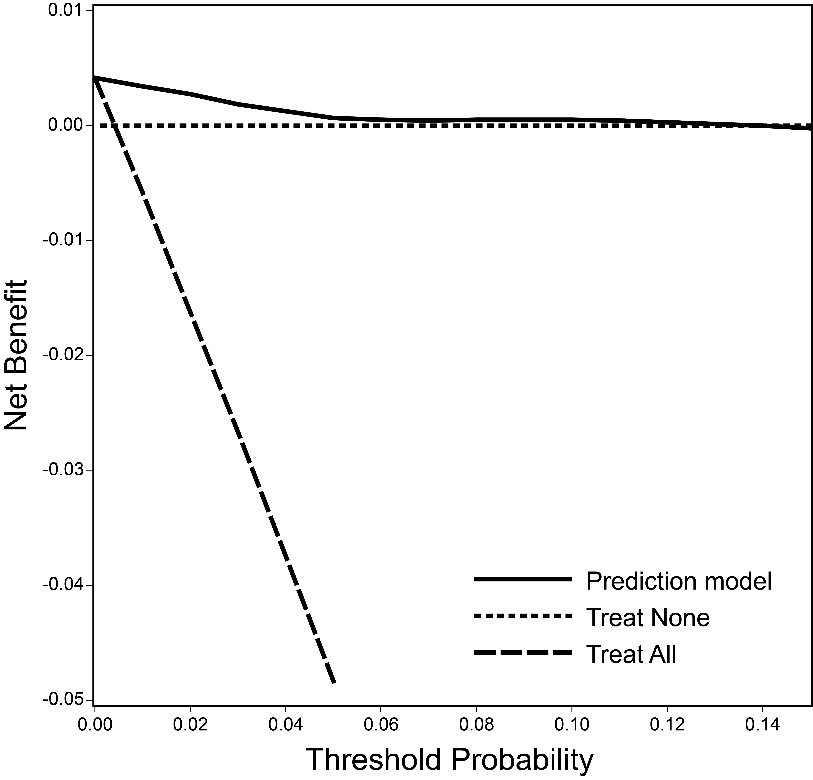
**

**Table S1. Demographic Characteristics of Patients Extracted from the Total Sample to Develop the Predictive Model.**

| **Variables** | **Cancer** | | **Non-cancer** | | **All** | |
| --- | --- | --- | --- | --- | --- | --- |
| **Training set** | **n=65** | | **n=16,275** | | **n=16,340** | |
| Age (years): mean (SD) | 65.9 | (6.5) | 55.2 | (10.2) | 55.2 | (10.2) |
| PSA slope (ng/mL/year): mean (SD) | 0.75 | (0.94) | 0.02 | (0.66) | 0.03 | (0.67) |
| Average PSA value (ng/mL): no. (%) |  |  |  |  |  |  |
| <1.5 | 1 | (1.5) | 13,147 | (80.8) | 13,148 | (80.5) |
| 1.5 – 2 | 2 | (3.1) | 1,302 | (8.0) | 1,304 | (8.0) |
| 2 – 3 | 12 | (18.5) | 1,070 | (6.6) | 1,082 | (6.6) |
| 3 – 4 | 16 | (24.6) | 427 | (2.6) | 443 | (2.7) |
| ≥4 | 34 | (52.3) | 329 | (2.0) | 363 | (2.2) |
| **Validation set** | **n=29** | | **n=6,976** | | **n=7,005** | |
| Age (years): mean (SD) | 64.7 | (7.3) | 55.2 | (10.3) | 55.2 | (10.3) |
| PSA slope (ng/mL/year): mean (SD) | 0.25 | (1.77) | 0.03 | (0.39) | 0.03 | (0.40) |
| Average PSA value (ng/mL): no. (%) |  |  |  |  |  |  |
| <1.5 | 0 | (0.0) | 5,614 | (80.5) | 5,614 | (80.1) |
| 1.5 – 2 | 0 | (0.0) | 589 | (8.4) | 589 | (8.4) |
| 2 – 3 | 4 | (13.8) | 462 | (6.6) | 466 | (6.7) |
| 3 – 4 | 10 | (34.5) | 167 | (2.4) | 177 | (2.5) |
| ≥4 | 15 | (51.7) | 144 | (2.1) | 159 | (2.3) |

PSA, prostate-specific antigen; SD, standard deviation

**Table S2. Results of Models including PSA slope or the Average PSA Value as Predictors of Continuous Variables (Traning set).**

| **Variables** | | **Age + PSA slope**  **(AUROC=0.85)** | | |  | **Age + Average PSA value**  **(AUROC=0.95)** | | |
| --- | --- | --- | --- | --- | --- | --- | --- | --- |
|  |  | Odds ratio | [95% CI] | P-value |  | Odds ratio | [95% CI] | P-value |
| Age | 1 year | 1.12 | [1.09, 1.15] | <0.001 |  | 1.11 | [1.08, 1.14] | <0.001 |
| PSA slope | 1 ng/mL/year | 1.24 | [1.12, 1.37] | <0.001 |  |  |  |  |
| Average PSA value | 1 ng/mL |  |  |  |  | 1.43 | [1.34, 1.53] | <0.001 |

CI, confidence interval; PSA, prostate-specific antigen
